# Decision thresholds for benefit-risk judgments: the new GRADE Working Group Guidance

**DOI:** 10.1590/S2237-96222026v35e20251401.en

**Published:** 2026-06-12

**Authors:** Erica Aranha Suzumura, Beate Jahn, Uwe Siebert, Patricia Coelho de Soarez

**Affiliations:** 1 Universidade de São Paulo, Faculdade de Medicina, São Paulo, SP, Brazil Universidade de São Paulo Faculdade de Medicina São Paulo SP Brazil; 2 UMIT TIROL - University for Health Sciences and Technology, Institute of Public Health, Medical Decision Making and Health Technology Assessment, Hall in Tirol, Tyrol, Austria UMIT TIROL - University for Health Sciences and Technology Institute of Public Health, Medical Decision Making and Health Technology Assessment Hall in Tirol Tyrol Austria; 3 ONCOTYROL - Center for Personalized Cancer Medicine, Division of Health Technology Assessment, Innsbruck, Tyrol, Austria ONCOTYROL - Center for Personalized Cancer Medicine Division of Health Technology Assessment Innsbruck Tyrol Austria; 4 Harvard T.H. Chan School of Public Health, Center for Health Decision Science and Departments of Epidemiology and Health Policy & Management, Boston, MA, United States Harvard T.H. Chan School of Public Health Center for Health Decision Science and Departments of Epidemiology and Health Policy & Management Boston MA United States; 5 Harvard Medical School, Mass General Brigham Center for Health Technology Assessment, Boston, MA, United States Harvard Medical School Mass General Brigham Center for Health Technology Assessment Boston MA United States

One of the publications issued in 2025 by the Grading of Recommendations Assessment, Development and Evaluation (GRADE) Working Group, GRADE Guidance 42, established a formal and transparent methodology for applying decision thresholds to determine the magnitude of the effects of a given intervention or technology on health outcomes^1^. Historically, judgments about the magnitude of effect (e.g., trivial, small, moderate or large) have been inconsistent and lacking in explicit justification. Decision thresholds are defined as predefined quantitative reference points adapted to specific outcomes. These thresholds serve as an additional tool among available methodologies^2^ to weigh the balance between desirable effects (benefits) and undesirable effects (risks or harms) in the processes of developing clinical guidelines and in health technology assessment studies.

## Selecting outcomes representing benefits and risks

The process begins with the selection and prioritization of health outcomes for which the effects of the use of a health technology will be assessed. Judgments regarding decision thresholds are made at the individual outcome level. Therefore, desirable outcomes (benefits) and undesirable outcomes (risks) most important to the health service user in the context of the benefit-risk assessment in question must be identified, ensuring that each is clearly defined (including how the outcome is measured/assessed and the time to measurement)^3^. The number of outcomes should be manageable in the context of the assessment (typically up to seven)^1^.

## Approaches for defining decision thresholds

GRADE suggests two main approaches and a step-by-step process for defining decision thresholds in judgments about the magnitude of benefits and risks. The first approach is based on generic coefficients derived from a methodological study conducted and published by the GRADE Working Group^4^. The second is based on expert opinion. It is important to note that these two approaches can be combined to verify the agreement of the decision thresholds defined through the different approaches^1^.

### Approach 1: based on generic coefficients

#### Methodological principle

The methodology for establishing decision thresholds is based on the expected utility theory. Utility values (ranging from 0, for the state of death, to 1, for full health) or its complement, disutility (1 - utility), serve as a basis for weighting the value of outcomes in such a way that they reflect the importance of that outcome in the population of interest^5^^), (^^6^. Utility values can be elicited through direct methods - such as the standard gamble method, time trade-off, or visual analog scale - or indirect methods - such as generic questionnaires for quality of life assessment (e.g., SF-6D, EQ-5D or HUI-3)^7^.

#### Identifying utility

Each outcome should be weighted by its respective utility value, which reflects the importance and preference for that outcome^5^^), (^^6^. The closer the utility value of an outcome is to 0, the more important that outcome is considered in decision-making. Ideally, utilities should be obtained from systematic reviews or primary studies using direct or indirect preference elicitation methods^7^. If neither of these options is feasible, utility determination can be done through consultation with experts, groups responsible for developing guidelines or external stakeholders, such as health service user representatives^8^.

#### Calculating decision thresholds

The GRADE Working Group uses four categories of effect magnitude: trivial or none, small, moderate and large^1^. Three decision thresholds must be determined for each outcome, separating the four magnitude of effect categories. The decision thresholds are expressed as absolute risk difference per 1,000 people.

Threshold calculation is done by applying linear regression equations to the disutility value (or 1 - utility) associated with the outcome. This ensures that the resulting decision thresholds are scaled according to the perceived importance of the specific outcome. The GRADE Working Group provides an online calculator for calculating decision thresholds from utility or disutility values (https://fmup.shinyapps.io/dt_calc)^1^. Thresholds can be calculated manually using the following regression equations, which employ generic coefficients.

- Decision threshold distinguishing between “trivial or no” effect vs. “small” effect (decision threshold_trivial/small_):

Decision threshold=0.073-(0.061 x disutility)

- Decision threshold distinguishing between “small” effect vs. “moderate” effect (decision threshold_small/moderate_):

Decision threshold=0.180-(0.146 x disutility)

- Decision threshold distinguishing between “moderate” effect vs. “large” effect (decision threshold_moderate/large_):

Decision threshold=0.338-(0.271 x disutility)

An outcome associated with a high disutility value (e.g., 0.9) will have lower decision thresholds compared to an outcome with low disutility (e.g., 0.2). This implies that, for a more important outcome, a smaller absolute risk difference is needed to categorize the magnitude of effect as “large” than for a less important outcome.

### Approach 2: based on expert opinion

This alternative relies on direct consultation with experts, decision-makers or other stakeholders. Experts are asked to explicitly state the absolute risk difference per 1,000 people that, in their judgment, separates the magnitude of effect categories for a specific outcome^1^. While simpler to implement, this approach must be carefully managed to mitigate potential cognitive biases and should be carried out without knowledge of the actual magnitude of the intervention effect (meta-analysis result)^1^. Because this approach involves direct determination of decision thresholds, obtaining utility values can be ignored when using this approach.

However, the GRADE Working Group encourages adoption of a combined approach, in which decision threshold sets are calculated using the linear regression equations presented above (Approach 1) and obtained through expert opinion (Approach 2). This allows decision-makers to compare decision threshold sets, and any significant discrepancy requires explicit discussion and transparent justification for the final selection of thresholds used in decision-making^1^.

#### Identifying the magnitude of effect

Once the decision thresholds have been defined, the summary effect of the evidence synthesis (i.e., the meta-analysis result) is compared or positioned in relation to these thresholds. This comparison is fundamental for determining and classifying the magnitude of the effect as trivial/none, small, moderate or large. For this assessment to be done correctly, the meta-analysis result must be expressed as the absolute risk difference per 1,000 people. Previous publications have provided instructions for calculating the absolute risk difference from the relative risk or time-to-event measures^9^^), (^^10^.

Two examples of decision thresholds calculated based on generic coefficients using the online calculator (https://fmup.shinyapps.io/dt_calc/) are illustrated in this paper (Figure 1). When assessing the outcome of deep vein thrombosis, the intervention demonstrated an absolute risk difference of -70 events per 1,000 people in relation to the comparator. Based on its associated disutility of 0.42^11^, the following thresholds were established for categorizing the magnitude of the effect: 47 events (threshold_trivial/small_), 119 events (threshold_small/moderate_) and 224 events (threshold_moderate/large_) per 1,000 people. Considering these limits, the effect estimate fell within the small magnitude benefit range, since the reduction of 70 events per 1,000 people exceeded the trivial effect size threshold but remained below the moderate effect size threshold (Figure 1A).

**Figure 1:**
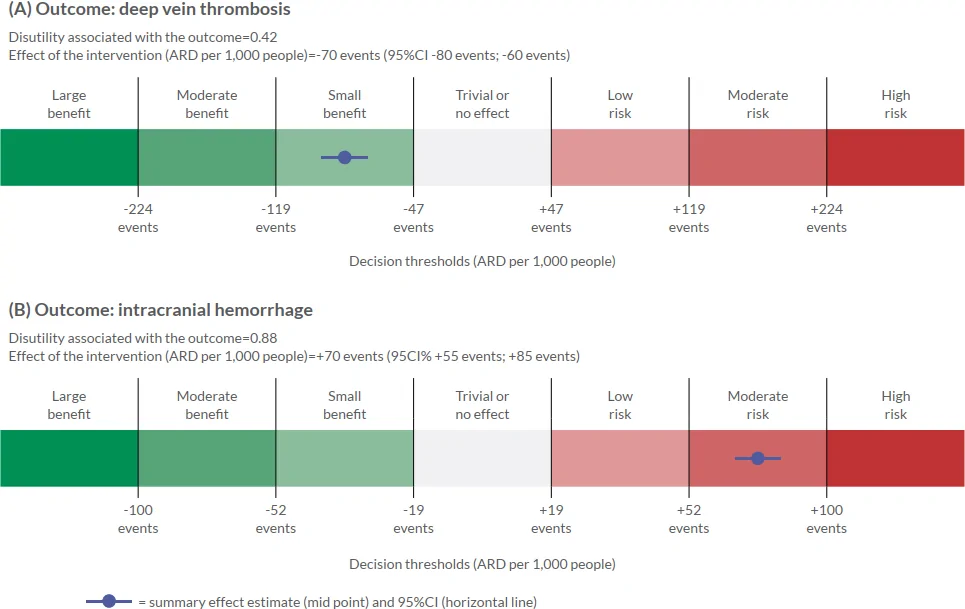
Decision thresholds (DT) calculated from the disutility values for (A) the deep vein thrombosis outcome and (B) the intracranial hemorrhage outcome, showing the grading of the summary effect estimates, expressed as absolute risk difference (ARD), and their respective 95% confidence intervals (95%CI) in relation to the thresholds that determine the magnitude of the effect (trivial/none, small, moderate or large).

Decision thresholds are presented in this paper for the intracranial hemorrhage outcome, the effect estimate of which demonstrated an absolute risk difference of +70 events per 1,000 people in relation to the comparator (Figure 1B). Considering its associated disutility of 0.88^11^, the thresholds calculated for categorizing the magnitude of effect were: 19 events (threshold_trivial/smal_), 52 events (threshold_small/moderate_) and 100 events (threshold_moderate/large_) per 1,000 people. In this scenario, the estimated effect of +70 events per 1,000 people was placed in the moderate magnitude risk range, situated between the small/moderate and moderate/large thresholds.

### Considerations

#### Overall balance and correlated outcomes

Although decision thresholds provide clarity for individual outcomes, overall judgment on the benefit-risk balance continues to be a key challenge, especially when outcomes are correlated (e.g., different manifestations of the same event). The GRADE Working Group recommends selecting independent outcomes or, if correlation is unavoidable, considering the outcome with the highest certainty of evidence to guide the judgment of balance^12^.

#### Incorporation of uncertainty

Although the effect of the technology under evaluation is represented by an effect estimate and its 95% confidence interval (95%CI), the decision thresholds for distinguishing the effect size are point values^1^. However, the confidence interval around the effect estimate can be used to judge certainty regarding the magnitude. For example, if the entire 95%CI of the absolute risk difference is in the “small” magnitude category, there is greater certainty in judging the effect size as “small”.

#### Limitations

Generic coefficients are based on hypothetical scenarios and may not be perfectly generalizable to all clinical contexts. Furthermore, obtaining standardized and robust utility values for all relevant health states continues to be a practical challenge^1^. The GRADE Working Group emphasizes that decision thresholds are not a substitute for the judgment of experts, methodologists and decision-makers, but rather an explicit tool to structure, inform discussion and support clinical guideline development processes and health technology assessment studies^1^. Therefore, expressing the balance between benefits and risks additionally as an incremental harm-benefit ratio remains important for decision-makers, service providers and service users in shared decision-making processes.

## Data Availability

The data are available within the body of the manuscript.
