# Supplementary material for: Decision thresholds for benefit-risk judgments: the new GRADE Working Group Guidance
Source: Epidemiol Serv Saude. 2026 Jun 12;35:e20251401. doi: 10.1590/S2237-96222026v35e20251401.en (PMC13686410; doi:10.1590/S2237-96222026v35e20251401.en)
Supplement: Translation

# Translation: Limiares de decisão para julgamentos sobre benefícios e riscos: a nova orientação do Grupo GRADE

**DOI:** 10.1590/S2237-96222026v35e20251401.pt

**Authors:** Erica Aranha Suzumura, Beate Jahn, Uwe Siebert, Patricia Coelho de Soarez

**Affiliations:** 1 Universidade de São Paulo, Faculdade de Medicina, São Paulo, SP, Brasil; 2 UMIT TIROL - University for Health Sciences and Technology, Institute of Public Health, Medical Decision Making and Health Technology Assessment, Hall in Tirol, Tirol, Áustria; 3 ONCOTYROL - Center for Personalized Cancer Medicine, Division of Health Technology Assessment, Innsbruck, Tirol, Áustria; 4 Harvard T.H. Chan School of Public Health, Center for Health Decision Science and Departments of Epidemiology and Health Policy & Management, Boston, MA, Estados Unidos; 5 Harvard Medical School, Mass General Brigham Center for Health Technology Assessment, Boston, MA, Estados Unidos

Uma das publicações de 2025 do Grupo de Trabalho Grading of Recommendations Assessment, Development and Evaluation (GRADE), Orientação GRADE 42, estabeleceu uma metodologia formal e transparente para a aplicação de limiares de decisão para determinar a magnitude dos efeitos de certa intervenção ou tecnologia sobre desfechos de saúde^1^. Historicamente, os julgamentos sobre magnitudes de efeitos (por exemplo, trivial, pequeno, moderado ou grande) têm sido inconsistentes e carentes de justificativa explícita. Os limiares de decisão são definidos como pontos de referência quantitativos predefinidos e adaptados a desfechos específicos. Esses limiares servem como ferramenta adicional entre as metodologias disponíveis^2^ para ponderar o balanço entre os efeitos desejáveis (benefícios) e indesejáveis (riscos ou danos) nos processos de desenvolvimento de diretrizes clínicas e nos estudos de avaliação de tecnologias em saúde.

## Seleção dos desfechos representando benefícios e riscos

O processo começa com seleção e priorização dos desfechos de saúde para os quais os efeitos do uso da tecnologia em saúde serão avaliados. Os julgamentos relativos aos limiares de decisão são feitos no nível do desfecho individual. Portanto, deve-se identificar desfechos desejáveis (benefícios) e indesejáveis (riscos) mais importantes para o usuário no contexto da avaliação benefício-risco em questão, garantindo que cada um esteja claramente definido (incluindo como o desfecho é mensurado/avaliado e o tempo até a mensuração)^3^. O número de desfechos deve ser manejável no contexto da avaliação (tipicamente até sete)^1^.

## Abordagens para a definição dos limiares de decisão

O GRADE sugere duas abordagens principais e o passo a passo para a definição dos limiares de decisão em julgamentos sobre a magnitude dos benefícios e riscos. A primeira abordagem é baseada em coeficientes genéricos derivados de um estudo metodológico conduzido e publicado pelo Grupo GRADE^4^. A segunda é com base na opinião de especialistas. É importante notar que as abordagens podem ser combinadas para verificar a concordância dos limiares de decisão definidos por meio das diferentes abordagens^1^.

### Abordagem 1: baseada em coeficientes genéricos

#### Princípio metodológico

A metodologia para o estabelecimento dos limiares de decisão está alicerçada na teoria da utilidade esperada. Os valores de utilidade (variando de 0, para o estado de morte, a 1, para saúde plena) ou o seu complemento, a desutilidade (1 - utilidade), servem como base para ponderar o valor dos desfechos de tal forma que reflitam a importância desse desfecho na população de interesse ^5^^), (^^6^. Os valores de utilidade podem ser elicitados por meio de métodos diretos - a exemplo do método de aposta padrão (*standard gamble*), troca de tempo (*time trade-off*) ou escala visual analógica - ou métodos indiretos - como os questionários genéricos para a avaliação de qualidade de vida (por exemplo, SF-6D, EQ-5D ou HUI-3)^7^.

#### Identificação das utilidades

Cada desfecho deve ser ponderado por seu respectivo valor de utilidade, o que reflete a importância e a preferência por esse desfecho^5^^), (^^6^. Quanto mais próximo de 0 estiver o valor de utilidade de um desfecho, mais importante esse desfecho é considerado na tomada de decisão. Idealmente, as utilidades devem ser obtidas a partir de revisões sistemáticas ou de estudos primários utilizando métodos diretos ou indiretos de elicitação de preferências^7^. Se nenhuma dessas opções for viável, a determinação das utilidades pode ser feita por meio de consulta a especialistas, grupos responsáveis pelo desenvolvimento de diretrizes, ou partes interessadas externas, como representantes de usuários^8^.

#### Cálculo dos limiares de decisão

O Grupo GRADE considera quatro categorias de magnitude de efeitos: trivial ou nenhum, pequeno, moderado e grande^1^. Para cada desfecho, três limiares de decisão devem ser determinados, separando as quatro categorias de magnitude de efeito. Os limiares de decisão são expressos como diferença de risco absoluto por 1 mil pessoas.

O cálculo dos limiares é feito aplicando-se equações de regressão linear ao valor de desutilidade (ou 1 - utilidade) associada ao desfecho. Isso assegura que os limiares de decisão resultantes sejam dimensionados de acordo com a importância percebida sobre o desfecho específico. O Grupo GRADE disponibiliza uma calculadora online para o cálculo dos limiares de decisão a partir dos valores de utilidade ou desutilidade (https://fmup.shinyapps.io/dt_calc)^1^. Os limiares podem ser calculados manualmente por meio das equações de regressão, a seguir, que empregam coeficientes genéricos.

- Limiar de decisão distinguindo efeito “trivial ou nenhum” vs. “pequeno” (limiar de decisão_trivial/pequeno_):

Limiar de decisão=0,073-(0,061 x desutilidade)

- Limiar de decisão distinguindo efeito “pequeno” vs. “moderado” (limiar de decisão_pequeno/moderado_):

Limiar de decisão=0,180-(0,146 x desutilidade)

- Limiar de decisão distinguindo efeito “moderado” vs. “grande” (limiar de decisão_moderado/grande_):

Limiar de decisão=0,338-(0,271 x desutilidade)

Um desfecho associado a um alto valor de desutilidade (por exemplo, 0,9) terá limiares de decisão menores em comparação com um desfecho de baixa desutilidade (por exemplo, 0,2). Isso implica que, para um desfecho com maior importância, uma diferença de risco absoluto menor é necessária para categorizar a magnitude do efeito como “grande” do que para um desfecho de menor importância.

### Abordagem 2: baseada em opinião de especialistas

Esta alternativa depende da consulta direta a especialistas, tomadores de decisão ou outras partes interessadas. Os especialistas são solicitados a declarar explicitamente a diferença de risco absoluto por 1 mil pessoas que, em seu julgamento, separa as categorias de magnitude de efeito para um desfecho específico^1^. Embora mais simples de implementar, essa abordagem deve ser gerenciada cuidadosamente para mitigar potenciais vieses cognitivos e deve ser realizada sem o conhecimento da magnitude real do efeito da intervenção (resultado da meta-análise)^1^. Como esta abordagem envolve a determinação direta dos limiares de decisão, a obtenção de valores de utilidade pode ser ignorada ao usar esta abordagem.

No entanto, o Grupo GRADE incentiva a adoção de uma abordagem combinada, na qual os conjuntos de limiares de decisão são calculados utilizando as equações de regressão linear apresentadas anteriormente (Abordagem 1) e obtidos por meio de opinião de especialistas (Abordagem 2). Isso permite aos tomadores de decisão comparar os conjuntos de limiares de decisão, e qualquer discrepância significativa exige discussão explícita e justificativa transparente para a seleção final dos limiares utilizados na tomada de decisão^1^.

#### Identificar a magnitude do efeito

Após a definição dos limiares de decisão, o efeito sumário da síntese de evidências (ou seja, o resultado da meta-análise) é comparado ou posicionado em relação a esses limiares. Essa comparação é fundamental para determinar e classificar a magnitude do efeito como trivial/nenhum, pequeno, moderado ou grande. Para que essa avaliação seja feita corretamente, o resultado da meta-análise deve estar obrigatoriamente expresso como diferença de risco absoluto por 1 mil pessoas. Publicações prévias apresentaram instruções para o cálculo da diferença de risco absoluto a partir do risco relativo ou medidas de tempo até o evento^9^^), (^^10^.

Dois exemplos de limiares de decisão calculados com base nos coeficientes genéricos usando a calculadora online (https://fmup.shinyapps.io/dt_calc/) foram ilustrados (Figura 1). Ao avaliar o desfecho trombose venosa profunda, a intervenção demonstrou diferença de risco absoluto de -70 eventos por 1 mil pessoas em relação ao comparador. Com base na desutilidade associada de 0,42^11^, foram estabelecidos os seguintes limiares para a categorização da magnitude do efeito: 47 eventos (limiar_trivial/pequeno_), 119 eventos (limiar_pequeno/moderado_) e 224 eventos (limiar_moderado/grande_) por 1 mil pessoas. Considerando esses limites, a estimativa de efeito posicionou-se na faixa de benefício de pequena magnitude, visto que a redução de 70 eventos por 1 mil pessoas superou o limiar de efeito de tamanho trivial, mas permaneceu abaixo do limiar de efeito de tamanho moderado (Figura 1A).

**Figura 1:** Limiares de decisão (LD) calculados a partir dos valores de desutilidade para os desfechos (A) trombose venosa profunda e (B) hemorragia intracraniana, mostrando o posicionamento das estimativas de efeito sumárias, expressas em diferença de risco absoluto (DRA), e respectivos intervalos de confiança de 95% (IC95%) em relação aos limiares que determinam a magnitude do efeito (trivial/nenhum, pequeno, moderado ou grande)

Apresentaram-se os limiares de decisão para o desfecho hemorragia intracraniana, cuja estimativa de efeito demonstrou diferença de risco absoluto de +70 eventos por 1 mil pessoas em relação ao comparador (Figura 1B). Considerando a desutilidade associada de 0,88^11^, os limiares calculados para a categorização da magnitude do efeito foram: 19 eventos (limiar_trivial/pequeno_), 52 eventos (limiar_pequeno/moderado_) e 100 eventos (limiar_moderado/grande_) por 1 mil pessoas. Nesse cenário, a estimativa de efeito de +70 eventos por 1 mil pessoas posicionou-se na faixa de risco de magnitude moderada, situando-se entre os limiares pequeno/moderado e moderado/grande.

### Considerações

#### Balanço geral e desfechos correlacionados

Embora os limiares de decisão forneçam clareza para desfechos individuais, um desafio central permanece sendo o julgamento global sobre o balanço benefício-risco, especialmente quando os desfechos são correlacionados (por exemplo, diferentes manifestações do mesmo evento). O Grupo GRADE aconselha a seleção de desfechos independentes ou, se a correlação for inevitável, considerar o desfecho com a maior certeza de evidência para orientar o julgamento do balanço^12^.

#### Incorporação da incerteza

Embora o efeito da tecnologia sob avaliação seja representado por estimativa de efeito e seu intervalo de confiança de 95% (IC95%), os limiares de decisão para distinguir o tamanho do efeito são pontuais^1^. No entanto, o intervalo de confiança em torno da estimativa de efeito pode ser utilizado para julgar a certeza em relação à magnitude. Por exemplo, se todo o IC95% da diferença de risco absoluto estiver na categoria de magnitude “pequena”, maior será a certeza em julgar o tamanho do efeito como “pequeno”.

#### Limitações

Os coeficientes genéricos são baseados em cenários hipotéticos e podem não ser perfeitamente generalizáveis para todos os contextos clínicos. Além disso, obter valores de utilidades padronizados e robustos para todos os estados de saúde relevantes continua sendo um desafio prático^1^. O Grupo GRADE enfatiza que os limiares de decisão não são um substituto para o julgamento de especialistas, metodologistas, e tomadores decisão, mas sim uma ferramenta explícita para estruturar, informar a discussão e apoiar processos de desenvolvimento de diretrizes clínicas e estudos de avaliação de tecnologias em saúde^1^. Portanto, expressar o balanço entre benefícios e riscos adicionalmente como uma razão de dano-benefício incremental continua sendo importante para tomadores de decisão, prestadores de serviços e usuários, nos processos de tomada de decisão compartilhada.

## Data Availability

Os dados estão disponíveis no corpo do manuscrito.
